# Therapeutics Based on microRNA: A New Approach for Liver Cancer

**DOI:** 10.2174/138920210791616671

**Published:** 2010-08

**Authors:** G Zhang, Q Wang, R Xu

**Affiliations:** Institute of Molecular Medicine, Huaqiao University & Engineering Research Center of Molecular Medicine, Ministry of Education, Quanzhou, Fujian, 362021, China

**Keywords:** Hepatocellular carcinoma, gene therapy, microRNA, hepatitis, hepatic ﬁbrosis, tumor suppressor genes, hepatic targeting vector.

## Abstract

Hepatocellular carcinoma (HCC) is a serious public health hazard. Polygenes involvement, accumulation of genetic and epigenetic changes and immune response of viral vector during gene therapy have resulted in the high mortality rate without marked change. To provide a safeguard for gene therapy and the feasibility for a clinical application, efforts have been focused predominantly upon constructing liver-targeted vector recently. MicroRNAs (miRNAs), a class of short endogenous RNAs, regulate the gene expression at the post-transcriptional level through imperfect base pairing with the 3′-untranslated region of target mRNAs. miRNAs, especially the liver-specific miRNA: miR-122, have multiple functions in liver development and abnormal expression of miRNAs could lead to liver diseases. Altered miRNA expressions have been observed in HCCs, viral hepatitis and hepatic ﬁbrosis. The different expression profiles of miRNAs in HCC suggest that miRNAs may serve as either novel potential targets acting directly as oncogenes or therapeutic molecules working as tumor suppressor genes. Moreover, the abundance in general and liver specificity in particular, all together make them attractive to be considered as elements for hepatic specific targeting viral vector. This review describes recent progress in miRNA investigation on liver associated for better understanding the relationship between miRNA and liver cancer in order to raise prospects for therapy.

## INTRODUCTION

Hepatitis, hepatic fibrosis and hepatocellular carcinoma (HCC) are the main liver diseases, threatening the health of human beings. Especially, HCC is one of the most common cancers worldwide and among the leading causes of cancer-related death [1] arises in the setting of chronic liver diseases, mostly related to viral hepatitis B and C [2-4] and cirrhosis [5]. As other malignant diseases, polygenes involvement coupled with accumulation of genetic and epigenetic changes [6-8] contribute to high mortality rate without marked changes, although unflagging effort has been devoted to study the molecular pathogenesis of HCC.

Many factors including exposure to hepatitis viruses [9-11], foodstuffs contaminated with aﬂatoxin B1(AFB_1_) [12], heavy alcohol intake [13], nonalcoholic fatty liver disease [14], oral contraceptives [15] and hemochromatosis [16] can lead to HCC. However, the exact pathophysiology of HCC is poorly understood. Underlying liver dysfunction a predisposing condition for HCC is the only thing we can confirm [5]. As a matter of fact, the predisposing condition for HCC dramatically changes the cellar signaling pathways, many genetic and epigenetic aberrations and the corresponding alterations in molecular pathways have been observed in the process of HCC. The involved pathways are depicted as follows: (a) activation of the Wnt/Frizzled/β-catenin pathway through mutations in β-catenin as well as up-regulation of upstream elements, such as Frizzled receptors [17-21], (b) alteration of the MAPK signaling pathway through HBV or HCV infection [22,23], (c) activation of the JAK/STAT pathway through inactivation of JAK-binding proteins [24-26], (d) inactivation of the tumor suppressor gene p53 through gene mutation and posttranscriptional interaction with viral proteins as well as oxidative stress [27-29], (e) alteration of the tumor suppressor retinoblastoma (pRb Pathway) and p16INK4 genes through mutations or promoter methylation [19-21], and (f) alteration of the transforming growth factor-β pathway [19-20].

So far, many HCC-related oncogenes, including *AFP*, *RAS*, *c-FOS*, *c-JUN*, *RHO*, *TGF-a*, *HGF*, *CerbB2*, *HER-2*, *HER-2/neu*, *NEU*, *NGL*, *MDM2*, *MMP*, and *IGF-*⫽ , have been found. The abnormal expression of these genes with regard to a lasting cell proliferation results in carcinogenesis ultimately [30].

There is a long way to go in fighting against HCC. Among all the therapies available for HCC, the surgical resection and liver transplantation are currently best curative options to treat liver cancer. The high frequency of tumor recurrence and metastasis after curative resection is the major obstacle in the process of HCC treatment. Statistics show that the survival rate of patients who have had a resection is 30% to 40% at 5 years, postoperatively [10]. Chemotherapy and radiotherapy, the two conventional therapies applied in the treatment of cancer, also get an unfavorable score because of the resistance of HCC. Moreover, occurrence of HCC often coupled with liver dysfunction, leads to restrict the use of conventional chemotherapeutics as there is more or less non-selective toxicity with significant systemic side effects [31]. Viral vectors, for example, recombinant adeno-associated virus (rAAV), mediated gene therapy which is targeting liver by hydrostatic pressure injection, is considered to be the appealing approach for liver disease as it is quite effective, associated with higher infectious rate and prolonged expression. However, immune response induced by viral vector coupled with the expression of heterologous gene in unwanted tissues or cells could shut down the function gene expression [30]. Other experimental treatment approaches, including hormonal therapy, biologic and biochemical therapy [32-36], and molecularly targeted therapy [37-40], are still needed to be further vertified in clinical application. There is an urgent need to develop novel treatments for recurrent and advanced HCC. 

A major endeavor to develop novel treatments should comprise the use of molecular profiling to characterize tumors and provide accurate predictions as well as potential therapeutic targets during the process of HCC. miRNAs, an abundant class of endogenous, small, noncoding RNAs, 19~25 nucleotides, can regulate the expression of protein-coding genes at the posttranscriptional level through imperfect base pairing with the 3′-untranslated region (3′-UTR) of target mRNAs. After the discovery of the first miRNA in the roundworm *Caenorhabditis elegans* by *Victor Ambros *[41], these short regulatory RNAs have been verified to be an abundant class of RNAs in plants, primates, rodents, birds, fishes, worms and flies ( http://microrna. sanger. ac.uk/). Large DNA viruses have also been found to carry miRNA genes: 5 in Epstein–Barr virus, 12 in Kaposi sarcoma-associated herpesvirus, 9 in mouse g-herpes-virus 68, and 9 in human cytomegalovirus [42]. miRNAs are post-transcriptional regulators of genes involved in the developmental timing, signaling pathways, apoptosis, metabolism, carcinogenesis and brain development [43]. Accumulating evidences have addressed that miRNAs are also involved in oncogene and tumor suppressor pathways [44-47]. Aberrant expression and structural alterations of miRNA genes have been found in a variety of tumor types [48-56].

miRNAs in liver, the regulators of genes, wear different expression profiles from nondiseased livers to that among the patients with HCC including those with cirrhosis and hepatitis infection [57-59]. The different expression of miRNAs between the normal liver and diseased liver might lead to a novel direction, which is helpful not only for diagnosis, but also for novel therapeutic targets in liver cancer therapy.

## miRNA IN LIVER CANCER BIOLOGY

**Fig. (1). Biogenesis and Regulation of miRNA.**
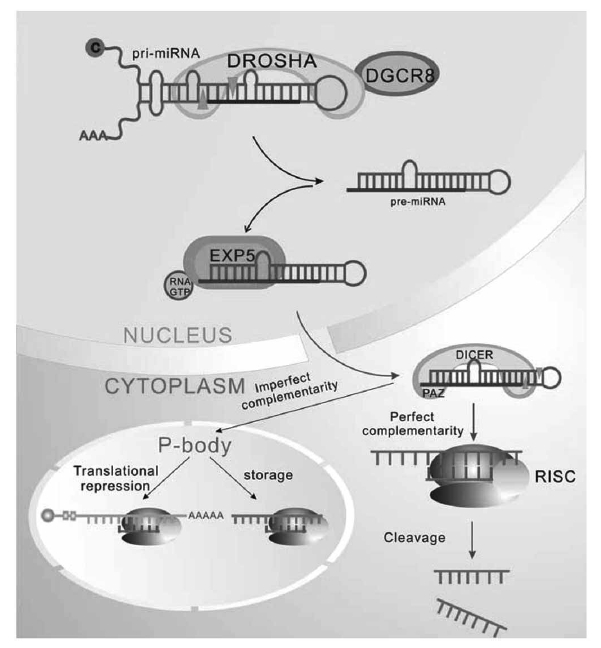
Transcription from the miRNA gene in the nucleus, the pri-miRNA is then cleaved by Drosha and DGCR8, producing a precursor molecule (pre-miRNA). With the help of Exportin-5 and Ran-GTP, the pre-miRNA is transported to the cytoplasm. In cytoplasm, the pre-miRNA is cleaved by the ribonuclease Dicer to generate a short RNA duplex. The mature single-stranded miRNA is incorporated into the RNA-induced silencing complex (RISC) to carry out the regulation function. The regulation mechanism conducting by miRNA dependents on the degree of complementarity between the 3′-UTR region of the target mRNA and this so called ‘seed region’ in the 5′-end of the miRNA, If there is perfect complementarity, then the mRNA is cleaved by RISC. If there is imperfect complementarity, regulation is carried out by repression of translation in P-body.

As extensively reviewed, the regulation of microRNA biogenesis (Fig. **1**) contributes to ﬁne-tuning of the cellular phenotype, including proliferation, cell signaling, and apoptosis; Undoubtedly, miRNAs involve in liver cancer biology.

### miRNA Expression Profiles in HCC

Increasing evidence indicated that aberrant expression of miRNAs plays an essential role in the pathogenesis of cancer, either by controlling the expression of known protein-coding genes, or by interacting with oncogenes or tumor suppressors [49]. Therefore, the expression profiles of miRNAs should play important roles in identifying their exact function in liver cancer biology, which may provide valuable information for diagnosis, classiﬁcation, progression, and even strategy for therapy [60].

A study based on the miRNA expression proﬁles in 25 pairs of HCC and adjacent non-tumorous tissue (NT) using a human miRNA microarray suggests that miRNA may potentially serve as a diagnostic tool of HCC. By comparing miRNA expression among HCC tissues versus the corresponding non-cancerous liver tissues, 30 miRNAs (Table **1**) had statistical differences with 3 miRNAs (Table **1**) signiﬁcant up-regulation and 5 miRNAs signiﬁcant down-regulation in HCC, respectively [57]. The accuracy of prediction in the samples as HCC or NT reached 97.8% (45/46) by using support vector machine algorithms based on these data provided above. Another study on the miRNA expression in 10 pairs of HCC and adjacent NT from 10 non-viral hepatitis patients, using a mammalian miRNA microarray containing whole human mature and precursor miRNA sequences disclosed that 15 miRNAs exhibited higher expression and one miRNA demonstrated lower expression in the HCC samples than in the NT samples, respectively [61]. Besides, it is very amazing to find that among a total of 18 miRNAs identiﬁed valid expression only in HCC samples, with 6 only in NT samples [61]. *Jiang* also showed that 16 miRNAs including miR-199a, miR-21, miR-223, and miR-150 were differentially expressed in the tumor compared with adjacent benign liver with 7 miRNAs up-regulation and 5 miRNAs signiﬁcant down-regulation (Table **1**) [58]. The potential use of miRNA profiling in subtyping human cancers to provide more accurate prognosis and prediction of response to therapy are illustrated in the article by *Ji*, they found that the expression of miR-26a and miR-26b in nontumor liver tissue which is higher in women than in men, was reduced in a subgroup of samples from patients with liver cancer during their study. Furthermore, patients with reduced miR-26 expression had significantly reduced survival during a 6-year period but were more likely to have a response to adjuvant therapy with interferon alfa [62]. However, the expression pattern of certain miRNAs has not presented the same among these reports. Maybe it is due to the differences in samples, testing methods and even the non-identical analytical methods. Nevertheless, the expression of hsa-miR-195 is down-regulation in some studies [57], while up-regulation in others [61].

In addition, the expression proﬁles of certain miRNAs can also characterize the metastasis of HCC [67-68]. By examining the miRNA expression profiles of 482 cancerous and noncancerous specimens from radical resection of 241 patients with HCC, *Budhu* built a unique 20-miRNA metastasis signature that could significantly predict primary HCC tissues with venous metastases from metastasis-free solitary tumors with 10-fold cross-validation [68]. *Zhang* found that up-regulated miR-143 also enhances hepatocarcinoma metastasis by repressing fibronectin expression [67]. Moreover, recent studies have documented that miRNAs may serve as either novel potential targets acting directly as oncogenes [69] or therapeutic molecules working as tumor suppressor genes [70]. Therefore, methods for liver cancer classification and therapy based on miRNAs′ unique sensitivity and specificity would prove to be very high-efficiency, succinct and rapid.

### Liver Associated miRNAs and p53 Tumor Suppressor Network

The aberrant expression of miRNAs in liver cancer may be attributing to the modulation of cancer-associated transcription factors. Nowadays, there is a clearer picture emerging from the miRNA processing [71-73]. What develops our interest is the relationship between miRNA processing and p53 tumor suppressor networks [74-77]. The p53 protein is a transcription factor that regulates multiple cellular processes in tumor development, either by regulating mRNA directly or by regulating miRNA indirectly. Therefore, the relationship between p53 and miRNA processing is of prime importance in the understanding of tumorigenesis. 

By now, accumulating studies have addressed that miRNAs are the components of tumor suppressor pathways. An interesting case is represented by miR-34 family. miR-34 family are direct transcriptional targets of p53, whose induction by DNA damage and oncogenic stress depends on p53 both *in vitro* and *in vivo *[78,79]. Study carried out by *Song* further indicated that miR-192 may be another miRNA candidate that is involved in the p53 tumor suppressor network with significant effect on cell cycle control and cell proliferation [75]. To identify the regulation mechanisms in p53-associated miRNA processing, *Suzuki* showed that p53 facilitates the processing of primary miRNAs to precursor miRNAs by interacting with the Drosha processing complex through the association with DEAD-box RNA helicase p68 [71]. 

In turn, miRNA can also directly regulate p53. *Le* firstly demonstrated that miR-125b as a negative regulator of p53 represses p53 protein levels in a manner dependent on its binding site in the p53 3′ UTRs [76]. The conclusion reached by the a fact that overexpression of miR-125b represses the endogenous level of p53 protein and suppresses apoptosis in human neuroblastoma cells, while knockdown of miR-125b elevates the level of p53 protein and induces apoptosis in human lung fibroblastsin. *Fornari* further showed that miR-122 can also directly regulate p53 by influencing p53 protein stability and transcriptional activity [80]. As the details of these and other miRNA biogenesis and regulatory processes unravel, the coming years promise to be an exciting time in miRNA-based liver cancer research.

## miRNAs AND VIRAL HEPATITIS

Viral hepatitis, the major preventable cause of HCC, is a significant medical and public health concern not only in China, but also throughout the world. It causes signiﬁcant morbidity and mortality [81]. Viral hepatitis is caused by infection with at least five distinct viruses, of which the three most commonly identified in the United States are hepatitis A virus (HAV), hepatitis B virus (HBV), and hepatitis C virus (HCV) [82]. Viral hepatitis can be very serious. By now, nearly 2 billion people ever infected with the hepatitis B virus (HBV) worldwide [83], and 350 million suffering from chronic HBV infection accounting for 320 000 deaths per year [84]. As for viral hepatitis C, an estimated 3% of the world’s population—more than 170 million people — are infected by HCV, resulting in 10,000 to 20,000 deaths a year in the United States [85]. Currently, therapeutics available for viral hepatitis, including Interferon alpha (IFN-α), Peginterferon-alpha-2a (PEG IFN-α) and Ribavirin still could not control the disease completely, Furthermore, both interferon or ribavirin are expensive and often cause severe side effects, limiting their broader use and underscoring a novel therapeutics to improve treatment outcomes.

Development of novel therapeutics should be based on the clear understanding of the exact mechanism in transcriptional activity and replication activity of hepatitis virus, especially HBV and HCV. As hoped for, such studies have drawn much attention recently. A growing body of evidence has documented that epigenetic changes may be relevant to occult HBV infection [60, 86]. Further study suggests that methylation at HBV cccDNA island 2 might be correlated with the impaired replication activity of HBV cccDNA, which provides further evidence that methylation of hepatitis B virus covalently closed circular DNA may modulates HBV replication [87]. 

In addition, there is a clear evidence to confirm that miRNAs also participate in the regulation activity of HBV. Data based on computational approaches showed that one viral mRNA was found to be targeted by the viral miRNA indicating that HBV may use viral miRNAs to regulate its own gene expression [88]. *Uprichard* further demonstrated that HBV replication and gene expression can be strongly inhibited by virus specific siRNA treatment [89], setting light on the use of RNAi in HBV gene therapy. However, the high sequence specificity of siRNAs, combined with prolonged treatment, promoted the emergence of siRNA-resistant virus variants. An improved study by using artificial miRNA (amiRNA) expression vector based on the murine miR-155 sequence shows that amiRNA could effciently suppress the expression and replication of HBV *in vitro* without the emergence of siRNA-resistant virus variants [90]. Another case in point is that by using Pol II promoter cassettes that transcribe anti-HBV primary miRNA (pri-miR-122) and pri-miR-31 shuttles, HBV replication is inhibited efficiently both *in vitro *and* in vivo *[91].

HCV is an enveloped, positive-strand RNA virus of the Hepacivirus genus with a genome of about 9.6 Kb encoding a polyprotein of approximately 3,000 amino acids [92]. HCV exhibits considerable genetic diversity, but the HCV 5′non-coding region (NCR) plays an important role in viral replication and translation activity is highly conserved. A recent study aimed at integrating RNA structure and functional analysis of the 5′NCR of HCV suggested that the 5′NCR domain I plays an important role in RNA translational efﬁciency [93]. *Liu’s* study addressed that cyclophilin A (CyPA), a cellular chaperone with peptidylprolyl-cis-trans-isomerase activity facilitates the replication of HCV RNA by forming specialized membrane structures through a recruiting mechanism [94].

Furthermore, progress in HCV investigation on siRNA and miRNA associated indicated, that RNA interference (RNAi) may be a promising therapeutic entity for viral infections. Since, the HCV genome is a single-stranded RNA that functions as both a template for transcription and template for a negative strand replication intermediate, it is a prime candidate for RNAi. Especially, the internal ribosome entry site (IRES) locating at the 5′noncoding region of the viral genome, the highly conserved sequence and important roles in translation make it as an ideal target for RNAi, which has been documented in many studies [95-98]. 

As a potential therapeutic entity for viral infections, miRNA will never be inferior to siRNA, if not better. So far, there is still no evidence to confirm that HCV could utilize self-coding miRNAs to regulate its own gene expression, but miRNAs from the host cells may play an essential role in the regulation activity of HCV. A charming case in point is represented by miR-122, a liver-specific miRNA. miR-122 has been addressed to facilitate the replication of HCV by targeting the viral 5′non-coding region [99]. The conclusion reached by the fact that HCV RNA can replicate in Huh 7 cells, which express miR-122, but not in HepG2 cells, which do not express miR-122 [99]. Further study [100] carried out by the same team reveals an important fact that the location of the miR-122 binding site in the Hepatitis C Virus RNA Genome indicates its effect on gene regulation as insertion of the HCV miR-122 binding sites into the 3′NCR of a reporter mRNA leads to downregulation of mRNA expression [100], while miR-122 interacts with the 5′end of the hepatitis C virus RNA genome, resulting in increased viral RNA [99]. 

In addition, miR-122 can indirectly facilitate HCV replication by down-regulation of heme oxygenase-1 (HO-1) expression [101], HO-1 can suppress Hepatitis C Virus replication [102]. Therefore, down-regulation of miR-122 and up-regulation of HO-1 may be new strategies for anti-HCV intervention and cytoprotection. At the same time, translation of HCV RNA is also certainly correlated with miR-122. Sequestration of miR-122 in liver cell lines strongly reduces HCV translation, whereas addition of miR-122 stimulates HCV translation in liver cell lines and the non-liver HeLa cells [103]. 

Among other miRNAs, miR-199a^*^, another liver-specific miRNA with a sequence similar to the HCV genome has been identified as a potential inhibitor of HCV replication [104]. Different from miR-122, overexpression of miR-199a^*^ inhibited HCV genome replication, inhibition of miR-199a^*^; however, accelerated viral replication.

## miRNAs AND HEPATIC FIBROSIS

Liver ﬁbrosis characterized by an activation of hepatic stellate cells (HSCs) is another cause accounting for HCC. HSCs are the principal liver cells that promote hepatic ﬁbrosis [105]. Normally, residing in the space of Disse, HSCs can be activated by inﬂammation associated with inﬂammatory cytokines—including tumor necrosis factor α (TNF-α), IL-6, CC-chemokine ligand 2/monocyte chemoattractant protein (MCP)-1, interleukin (IL)-1, and TGF-α [106]. While some of inﬂammatory cytokines exert its effectiveness in hepatic ﬁbrosis by inflencing the expression of liver ﬁbrosis associated miRNAs [107,108]. Nowadays, the point of view that liver ﬁbrosis can be reversal has been generally accepted [109] provided that the activated HSCs switch to a more quiescent HSC. Here we capture key advances in miRNA-based liver ﬁbrosis study in order to raise potentials for therapy.

HSCs contain bunches of vitamin A-riching lipid droplets, while activated HSCs lose cytoplasmic lipid droplets and trans-differentiate to proliferative, and ﬁbrogenic myoﬁbroblasts play an essential role in the formation of liver ﬁbrosis [105]. A recent study based on down-regulation of miR-27a and 27b, two miRNAs that over-expressed in primary culture-activated rat HSCs documented that the culture-activated rat HSCs switch to a more quiescent HSC phenotype, with restored cytoplasmic lipid droplets and decreased cell proliferation [110]. Different from miR-27a and 27b, miR-29a and 29b are often downregulated in the formation of liver ﬁbrosis, *in vitro* study showed that miR-29 as novel antiﬁbrogenic mediators can repress collagen synthesis [111]. Although, it is the only example available, it may raise the curtain at the age of miRNA –based liver ﬁbrosis therapy.

## LIVER-SPECIFIC miRNA SIGNATURES IN LIVER DISEASE

Certain miRNAs are expressed ubiquitously, whereas others are expressed in a highly tissue-specific manner [112]. miR-122 accounting for 70% of the total miRNA population, is one of the birds specifically and abundantly expressed in the liver with undetected in all other tissues [112,113]. 

There is a long way to identify the liver-specific miRNA:miR-122. As the precursor for miR-122 was discovered in 1989. Furthermore, systematic cloning and sequencing of small RNAs prepared from different mouse tissues revealed that miR-122 was an abundant miRNA in the liver. The further study addressed that miR-122 is found in mouse, woodchuck and human livers, in human primary hepatocytes, and in cultured liver-derived cells, such as mouse Hepa 1-6 cells and human Huh7 cells [114]. Studies based on computational tools showed that the putative miR-122-target genes involved in cellular stress response [113], hepatocarcinogenesis [65, 115] and viral infection [99, 103], which were further experimentally conﬁrmed in cultured hepatocytes.

miR-122 plays an important role in the pathology of various diseases, including cancer, infection and metabolism disease. Analysis of RNAs from 20 human HCC samples showed that miR-122 was signiﬁcantly down-regulated in 50% of the tumors compared to non-malignant liver tissue from the same individuals [116], which is reconfirmed by others [117]. Further study shows that miR-122 also plays an important role in intrahepatic metastasis. Overexpression of miR-122 by a lentiviral vector (lenti-122) in metastatic Mahlavu and SK-HEP-1 cells signiﬁcantly reduced *in vitro* migration, invasion, and anchorage-independent growth as well as* in vivo* tumorigenesis, angiogenesis, and intrahepatic metastasis in an orthotopic liver cancer model [118]. 

Moreover, the infection of the hepatitis C virus (HCV) was also dependent on the status of miR-122 expression [99, 103]. A study carried out by *Catherine L* showed that HCV RNA can only replicate in cells expressing miR-122, but the replication failed to function. The role of miR-122 in HCV RNA replication was confirmed by silencing miR-122 in Huh7 cells with a marketed loss of replicating result [99]. At the same time, translation of HCV RNA is also certainly correlated with miR-122. Sequestration of miR-122 in liver cell lines strongly reduces HCV translation, whereas addition of miR-122 stimulates HCV translation in liver cell lines as well as in the non-liver HeLa cells and rabbit reticulocyte lysate. Further evaluating the role of miR-122 reveals that miR-122 stimulates HCV translation by enhancing the association of ribosomes with the viral RNA at an early initiation stage [103].

The overall importance of miR-122 in the regulation of metabolism has been elaborated through an antisense strategy speciﬁc to miR-122 so far [119]. Hepatic steatosis can be strikingly reduced in high-fat fed mice by silencing miR-122 in an antisense strategy based on a 2′-OMe phosphorothioate-modiﬁed oligonucleotide [119]. Similarly, silencing miR-122 also resulted in an increase in expression of several hundred genes, which were notably represented as putative miR-122 target genes, including those that are normally repressed in hepatocytes, as well as a decrease in expression of several genes, including those that are involved in cholesterol biosynthesis [119]. All these results argued for the importance of miR-122 in maintaining an adult-liver phenotype by regulating the expression of non-liver genes.

As mentioned above, Liver-specific miRNA (miR-122) plays very important roles in the pathology of various liver diseases, which also implies miR-122 might serve as a potential therapeutic target. For instance, therapeutics based on inhibiting miR-122 have proven to be efficient in inhibiting viral replication both *in vitro* and *in vivo* over the last several years [91, 120]. The importance in function [119] and convenience in regulation [120-123], all together make therapeutics based on miR-122 attractive to be used in the liver disease. One potential therapeutic application comes from the effect of miR-122 antagomir in high-fat fed mice to reduce hepatic steatosis, which may provide an interesting opportunity to treat patients with non-alcoholic steatohepatitis [119]. Another interesting application of miR-122 antagomir contributes in taking advantage of its effect on the down-regulation of adult-liver genes expression to generate *in vitro* a new attractive expandable cell source for hepatocyte transplantation that would feature stem/progenitor cell phenotype [119]; the third attracting application of miR-122 antagomir contributes in inhibiting viral replication and translation, which may provide an interesting opportunity to treat patients with viral infection [99, 103].

## miRNAs AS GENERAL ANTICANCER THERAPEUTICS 

miRNAs modulating gene expression through sequence complementarity can influence a series of biological processes, including differentiation, proliferation, apoptosis, angiogenesis, invasion and metastasis. As the deregulation of these very same processes is a hallmark of cancer, on one hand, it directly suggests that miRNAs may work as putative tumor suppressor genes or oncogenes, and on the other hand, it indirectly indicates that efforts for cancer therapy should be focused on these putative tumor suppressor genes or oncogenes. Increasing data have documented the possibility that miRNAs-based treatment may be as promising anticancer therapeutics [70]. Here, we summerize the evidence available in HCC.

To date, accumulating evidences have addressed that the putative tumor suppressor miRNAs may be the novel therapeutic entity for HCC, particularly for miR-26a [70], which is normally expressed at high levels in normal adult liver but dramatic downregulation in both human and murine liver tumors. A study based on miR-26a replacement by using AAV as delivery vector potently suppresses cancer cell proliferation and activates tumor-speciﬁc apoptosis *in vivo*, leading to dramatic suppression of tumor progression without toxicity, as miR-26a induces a G1 arrest in human liver cancer cells by downregulating cyclins D2 and E2 [70]. Similarly, in HCC, Osteopontin (OPN) is identiﬁed as one of the leading genes that promote the metastasis of HCC [124]. A recent study based on lentiviral vectors encoding microRNA against OPN reveals that silencing OPN can dramatically inhibits in both *in vitro* invasion and *in vivo* lung metastasis of HCCLM3 cells, even could suppress *in vitro* proliferation and *in vivo* tumor growth of HCCLM3 by interrupting MAPK pathway and NF-κB pathway [125], which suggests that OPN could be a hopeful target for the control of metastasis as well as HCC tumor growth and viral vector-mediated microRNA against OPN can be treated as a new therapeutics.

Among other putative tumor suppressor miRNAs, miR-101 [64, 126], miR-122 and miR-223 are of particular interest. The miR-101 is significantly down-regulated in the majority of cancer cell lines and cancer tissues examined. By targeting Mcl-1 [64], an antiapoptotic member of Bcl-2 family [127], and repressing the expression of the FOS oncogene [126], miR-101 not only suppresses colony formation *in vitro* and tumorigenicity* in vivo* but also sensitizes cancer cells to apoptosis induced by various chemotherapeutic drugs. Therefore, miR-101 could be a hopeful target for not only anticancer therapy but also a prognostic molecular marker for diagnose. The liver-speciﬁc miRNA, miR-122, could be detected as early as 12.5 days post-implantation and reaches a plateau immediately before birth [113], suggesting that miR-122 may play a critical role in liver development. Recent studies [65,118] showed that miR-122 is significantly down-regulated in liver cancer, which may function as tumor suppressor. By restoring miR-122 in metastatic Mahlavu and SK-HEP-1 cells, migration, invasion *in vitro* as well as tumorigenesis, angiogenesis, and intrahepatic metastasis *in vivo* are significantly inhibited [118]. Further study addressed that miR-122 inhibits hepatocellular carcinoma intrahepatic metastasis by modulating ADAM17 (a disintegrin and metalloprotease 17), a key component in angiogenesis. Besides, miR-122 can target the 3′-UTR of cyclin G1 (CCNG1) mRNA for its regulation. An inverse correlation between miR-122 and CCNG1 exists in primary liver carcinoma, further emphasizing the importance of miR-122 in HCC pathogenesis [65]. It is not more than a beginning. There are many novel miR-122 targets unidentified. A recent study by using miRNA-like siRNA expression vectors provides evidence that miR-122 can directly repress the Bcl-w protein level by targeting binding sites in the 3′-UTR [128]. Furthermore, ADAM10 (a distintegrin and metalloprotease family 10), serum response factor (SRF), and insulin-like growth factor 1 receptor (Igf1R) were all validated as targets of miR-122 [129]. The study carried out by Coulouarn emphasized miR-122 as a diagnostic and prognostic marker for HCC progression, such a point of view reached by the fact that loss of miR-122 results in an increase of cell migration and invasion and that restoration of miR-122 reverses this phenotype [130,131]. In addition, the loss of miR-122 is associated with liver-enriched transcription factors, such as HNF1A, HNF3A and HNF3B [130]. All these data suggest miR-122 plays a very important role in liver cancer, which is also an attractive therapy target for liver cancer. Similar to miR-122, miR-223 is also significantly down-regulated in HCC. Re-expression of miR-223 in HBV, HCV, and non-HBV non-HCV-related HCC cell lines revealed a consistent inhibitory effect on cell viability. Further study implicated that Stathmin 1 (STMN1) is a downstream target of miR-223. The substantial reduction in STMN1 protein was demonstrated upon restoration of miR-223 expression in HCC cell lines [132]. Therefore, miR-223 may represent a novel target in liver cancer therapy because it regulates STMN1, which is a good marker of the PTEN/PI3K path-way activity.

miRNAs, which are amplified or overexpressed in cancer could act as putative oncogenes or miRNAs that targets one or more tumor suppressor genes to inhibit the activity of an anti-oncogenic pathway. The miR-21 is one of the chief actors. Different from the putative tumor suppressor miRNAs mentioned above, miR-21 is always highly over-expressed in HCC [58, 61, 63, 117]. Inhibition of miR-21 in cultured HCC cells increased expression of the phosphatase and tensin homolog (PTEN) tumor suppressor, and decreased tumor cell proliferation, migration, and invasion [117]. Consistent with the results, an increase in tumor cell proliferation, migration, and invasion was observed in tumor cells transfected with precursor miR-21 [117]. Another study based on antisense oligonucleotides specific for miR-21 presented the idea that miR-21 played an important role in the maintenance of the malignant transformation of hepatocytes [133]. All these data showed that overexpression of miR-21 can contribute to HCC growth and spread by modulating PTEN expression. PTEN is a direct target of miR-21 and silencing miR-21 maybe a newly attractive therapeutics. Another interesting case in consideration is miR-221, similar as reported in other tumors [134], miR-221 is observed to be up-regulated also in HCC. Besides, the previously reported targets p27 and p57 [135], miR-221 can inhibit apoptosis by targeting Bmf, up-expression coupled with affecting multiple pro-oncogenic pathways indicate that miR-221 is a potential target for nonconventional treatment against HCC [136]. *In vivo* study based on a mouse model of liver cancer carried out by Pineau *et al.* addressed that miR-221 overexpression stimulates growth of tumorigenic murine hepatic progenitor cells by turely regulating DNA damage-inducible transcript 4 (DDIT4), a modulator of mTOR pathway [137]. Garofalo *et al*. further demonstrated that miR-221 could induce TRAIL resistance and enhance cellular migration through the activation of the AKT pathway and metallopeptidases by targeting PTEN and TIMP3 tumor suppressors [138].

To sum up the points, therapeutics based on miRNA is a potential therapy for HCC, even most of the possible pathways regulated by these miRNAs are still unclear. However, it will not blurr the possibility of treating miRNAs as general anticancer therapeutics, as strategies based on either silencing or restoring a miRNA in suppression of tumor progression, migration, invasion tumorigenesis, angiogenesis, and intrahepatic metastasis prove to be significantly effective. Moreover, not only silencing a miRNA in using antisense oligonucleotides specific but also restoring a miRNA in using viral vectors proved to be perfectly available. Here, we can boldly give prophesy that therapeutics based on miRNA will have an infinitely bright future.

## miRNAs AND VECTOR LIVER-TARGETING 

As stated, RNAi based on siRNA and miRNA could be one of the most promising avenues for the development of antiviral therapies and anticancer therapies. The success of RNAi in therapeutic application also depends on an efficient delivery system, which can support long-term siRNA production and continuous gene silencing. So far, the most powerful gene therapy vectors come from viruses with several steps of modification, as an ideal delivery system, gene therapy vector should be targeted specifcally such that they transduce target cells, while avoiding sequestration in other organs or toxicity from infection of unwanted cells. Considerable methods including transcriptional targeting [139], transductional targeting [140] and translational targeting [141], have been used for vector targeting. However, none is effective to all the vectors. Vector targeting by engineering the cassettes to contain miRNA target (miRT) elements that can then be recognized and regulated by endogenous cellular miRNAs has greatly attracted much attention. Here, we present the evidence available that miRNAs can be used for vector liver-targeting.

**Fig. (2). Eliminate the effects of potential contaminating from replication competent AAV by tissue-specific miRNAs.**
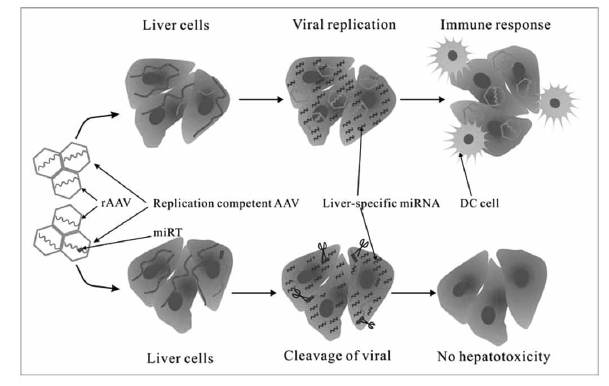
Incorporation into a packaging plasmid (green) of target sequences (red) recognized by liver-specific miRNAs (purple) ensures that the replication competent virus can’t replicate in liver cells.

Nowadays, an increasing evidence suggested that tissue-specific miRNAs play essential roles in regulating vector liver-targeting. One potential application comes from the impact of vector containing tissue-specific miRT elements in the specific tissue to shut down capsid genes expression, which provide an interesting opportunity to systematically reduce/eliminate the effects of potential contaminating from replication competent virus during virus vector-mediated gene therapy [142] (Fig. **2**). 

Another interesting application of tissue-specific miRNAs consist in taking advantage of its eﬀect on the down-regulation of transgene expression in hematopoietic lineages to escape immune response. Currently, one of the major barriers to stable gene transfer is the development of transgene-speciﬁc immunity [143] because of the direct expression of the transgene product within professional antigen-presenting cells (APCs) of the immune system [144]. Even tissue-speciﬁc promoters are used, immune reaction can still be observed. A recent study [145] based on lentiviral vectors encoding target sequences for hematopoietic specific mir-142-3p documented that, by preventing transgene expression in hematopoietic lineages while permitting high levels of expression in nonhematopoietic cells, miRNA regulation could enable stable gene transfer in the absence of an immune response [146]. The rational application was reconfirmed by others [146-148].

The third attractive application of tissue-specific miRNAs in vector liver-targeting comes from gene therapy, based on oncolytic viruses to reduce hepatotoxicity. By incorporating a casette that contains sequences complementary to the liver-speciﬁc miR-122 in the 3′UTR of the E1A gene, the recombinant adenovirus replicated normally in other cells but not in cells of hepatic origin [149-151]. Moreover, suicide gene therapy based on Ad vectors that mediate miR-122a-regulated HSVtk expression can be more safe and efficient by reducing hepatotoxicity perfectly [152]. 

As is known to all, miRNAs always signature in various diseases characterized by deregulation of miRNAs. Especially in cancer, oncogenic miRNAs are found to be ubiquitously expressed in normal tissues, but highly enriched in tumors, while tumor suppressor miRNAs specifically downregulated in cancer. Therefore, target elements to be tumor suppressor miRNAs responsive engineered within a vector can increase gene silencing in normal tissue, leaving gene expressing in cancer specially. For example, let-7, miR-143 and miR-145, as putative tumor suppressor miRNAs, are proved to be low expression in certain cancer cells [153]. The regulation based on either let-7 or miR-143 and miR-145 endows wild-type viral tumor-specific replication while eliminates undesirable replication and associated toxicity in normal cells [154,155]. All these data suggest that vector targeting regulated by miRNA could be not only tumor-specific but also few hepatotoxicity and immune response. It is therefore expected to be of a major utility for the generation of liver-targeted expression vectors.

## CONCLUSION AND PROSPECTIVE

Identification of miRNA function involved in liver cancer has provided an important knowledge regarding miRNA-tumor associated gene interactions and revealed many potential therapeutic targets. As documented in many studies, some miRNAs may function as oncogenes while others act as tumor suppressors. For oncogenic miRNAs, such as miR-21, a desirable therapeutic strategy, reduce their functions in cells. As for tumor suppress miRNAs, restoring that miRNA should provide the attractive outcome. 

Despite remarkable progress in miRNA-based therapy, many questions remain to be answered. The first nut for us to crack is that we should have a clear understanding of the exact mechanism to be responsive to miRNA regulation and the physiological function in the course of the life cycle. It is rarely known very little about the cellular circuits controlled by miRNAs in general and by cancer-associated miRNAs in particular. Efforts should be made to check the true frequency of mutations in miRNAs and in their target sequences. Besides, computational methods should be adopted to get a more comprehensive understanding of their mechanism of action. Only in this way, can we get a clearer picture of the role of miRNAs in human cancer. Different miRNAs are expressed at different copy numbers, the same miRNA is expressed at different copy numbers in different cell lineages. What interests us most is that the threshold copy of miRNAs must be reached to achieve appreciable gene silencing. However, there is less data available for reference. 

As one of the most promising avenues for the development of anticancer therapies, the success of miRNA in therapeutic application relies on an efficient delivery system. Nowadays, vector targeting is still one of the major barriers to achieve stable gene transfer. Gathering studies suggest vector targeting by engineering the cassettes to contain miRNA target (miRT) elements that could then be recognized and regulated by endogenous cellular miRNAs is very effective and versatile, although, much work remains to be done on the exact number of copies and the spacing elements between tandem copies of miRT elements, which will prove the most effcacious for vector targeting. The rAAV mediated gene therapy is considered to be the appealing approach for liver disease. Nevertheless, immune response induced by capsid synthesis in targeting tissues from residual contaminating replication competent AAV particles often shut down the function gene expression. To systematically reduce/eliminate the effects of potential contaminating rcAAV particles, our laboratory designed a novel AAV helper (pH22mir) with a microRNA binding cassette containing multiple copies of liver specific (hsa-mir-122) and hematopoietic specific (has-mir-142-3p) sequences to specifically control cap gene expression [142]. In the liver, 99.9% of capsid expression could be suppressed and no cap expression could be detected by the Western blot. Overall, it is difficult to overestimate the potential impact of the regulatory circuits of miRNAs in the liver, which may provide attractive targets for treatment of liver cancer.

## Figures and Tables

**Table 1.** miRNA Expression Profiles in HCC Compared with Normal Liver

miRNA Class	miRNA	Liver-tumor dysregulation							Special in HCC [61]
		Murakami(2006) [57]	Jiang(2008) [58]	Huang(2008) [61]	Ladeiro(2008) [63]	Su(2009) [64]	Gramantieri(2007) [65]	Huang(2009) [66]	
hsa-miR-18	miR-18	Up	Up						
	precursor miR-18	Up							
hsa-miR-21	miR-21		Up	Up	Up				
hsa-miR-33	miR-33		Up						
hsa-miR-101	miR-101		Down			Down			
hsa-miR-130	miR-130b		Up					Up	
	miR-130a						Down	Up	
hsa-miR-135	miR-135a		Up						
hsa-miR-139	miR-139		Down						
hsa-miR-150	miR-150		Down						
hsa-miR-199	miR-199a	Down	Down					Down	
	miR-199a*	Down	Down						
	miR-199b		Down						
hsa-miR-200	miR-200a	Down						Down	
	miR-200b		Down						
	miR-200c				Down				
hsa-miR-214	miR-214		Down						
hsa-miR-221	miR-221		Up					Up	
hsa-miR-223	miR-223		Down						
hsa-miR-301	miR-301		Up						
hsa-miR-224	miR-224	Up			Up			Up	
hsa-miR-125	miR-125a	Down						Down	
hsa-miR-235	miR-235			Down					
hsa-miR-22	miR-22			Up					
mmu-miR-126	miR-126			Up				Down	
	miR-126-3p			Up	Down				
hsa-let-7	let-7b			Up			Down	Down	
	let-7c			Up			Down	Down	
	let-7g			Up			Down	Down	
	let-7i			Up			Down	Down	
	let-7f			Up			Down	Down	
	let-7d			Up			Down	Down	
	let-7e			Up			Down	Down	
	let-7a-1			Up			Down	Down	
	let-7a-2			Up			Down	Down	
	let-7a-3			Up			Down	Down	
hsa-miR-124	miR-124a-2						Down	Down	
hsa-miR-132	miR-132						Down		
hsa-miR-136	miR-136						Down		
hsa-miR-141	miR-141						Down		
hsa-miR-142	miR-142						Down	Down	
hsa-miR-143	miR-143						Down	Down	
hsa-miR-145	miR-145						Down		
hsa-miR-150	miR-150						Down		
hsa-miR-155	miR-155						Down		
hsa-miR-181	miR-181a-1						Down		
	miR-181a-2						Down		
	miR-181c						Down		
hsa-miR-122	miR-122a						Down	Down	
hsa-miR-98	miR-98			Up					
rno-miR-352	miR-352			Up					
hsa-miR-195	miR-195	Down		Up				Down	
hsa-miR-523	miR-523								Yes
hsa-miR-34	miR-34a								Yes
rno-miR-146	miR-146								Yes
hsa-miR-121	miR-121								Yes
hsa-miR-526	miR-526a								Yes
hsa-miR-30	miR-30d								Yes
hsa-miR-146	miR-146b								Yes
hsa-miR-148	miR-148a								Yes
rno-miR-17	miR-17							Up	Yes
hsa-miR-215	miR-215								Yes
hsa-miR-192	miR-192								Yes
hsa-miR-93	miR-93								Yes
hsa-miR-107	miR-107							Up	Yes
hsa-miR-29	miR-29a								Yes
hsa-miR-103	miR-103								Yes
hsa-miR-146	miR-146a								Yes
hsa-miR-15	miR-15a							Down	Yes
hsa-miR-203	miR-203				Down				
hsa-miR-10	miR-10b				Up				
hsa-miR-222	miR-222				Up			Up	
hsa-miR-375	miR-375				Down				
hsa-miR-96	miR-96				Up				

## ABBREVIATIONS

HCC: Hepatocellular carcinoma
rAAV: Recombinant adeno-associated virus
miRNA: MicroRNA
UTR: Untranslated region
AFB_1_: Aflatoxin B_1_
NT: Nontumorous tissue
HAV: Hepatitis A virus
HBV: Hepatitis B virus
HCV: Hepatitis C virus
amiRNA: Artifcial miRNA
NCR: Non-coding region
CyPA: Cyclophilin A
IFN-α: Interferon alpha
PEG IFN-α: Peginterferon-alpha-2α
RNAi: RNA interference
IRES: Internal ribosome entry site
HO-1: Heme oxygenase-1
OPN: Osteopontin
ADAM17: A disintegrin and metalloprotease 17
CCNG1: Cyclin G1
ADAM10: A distintegrin and metalloprotease family 10
SRF: Serum response factor
Igf1R: Insulin-like growth factor 1 receptor
STMN1: Stathmin 1
miRT: miRNA target

## References

[1] Parkin DM, Bray F, Ferlay J, Pisani P (2005). Global cancer statistics, 2002. CA Cancer J. Clin.

[2] Bosch FX, Ribes J, Borras J (1999). Epidemiology of primary liver cancer. Semin. Liver Dis.

[3] Arbuthnot P, Kew M (2001). Hepatitis B virus and hepatocellular carcinoma. Int. J. Exp. Pathol.

[4] Bruix J, Boix L, Sala M, Llovet JM (2004). Focus on hepatocellular carcinoma. Cancer Cell.

[5] Aravalli RN, Steer CJ, Cressman ENK (2008). Molecular Mechanisms of Hepatocellular Carcinoma. Hepatology.

[6] Herath NI, Leggett BA, MacDonald GA (2006). Review of genetic and epigenetic alterations in hepatocarcinogenesis. J. Gastroenterol. Hepatol.

[7] Farazi PA, DePinho RA (2006). Hepatocellular carcinoma pathogenesis: from genes to environment. Nat. Rev. Cancer.

[8] Su H, Zhao J, Xiong Y, Xu T, Zhou F, Yuan Y, Zhang Y, Zhuang SM (2008). Large-scale analysis of the genetic and epigenetic alterations in hepatocellular carcinoma from Southeast China. Mutat. Res.

[9] Cramp ME (1999). HBV plus HCV=HCC?. Gut.

[10] Blum H (2005). Hepatocellular carcinoma: therapy and prevention. World J. Gastroenterol.

[11] El-Serag HB, Rudolph KL (2007). Hepatocellular carcinoma: epidemiology and molecular carcinogenesis. Gastroenterology.

[12] Soini Y, Chia SC, Bennett WP, Groopman JD, Wang JS, DeBenedetti VM (1996). An a?atoxin-associatedmutational hotspot at codon 249 in the p53 tumor suppressor gene occurs in hepatocellular carcinomas from Mexico. Carcinogenesis.

[13] Donato F, Tagger A, Gelatti U, Parrinello G, Boffetta P, Albertini A, Decarli A, Trevisi P, Ribero ML, Martelli C, Porru S, Nardi G (2002). Alcohol and hepatocellular carcinoma: the effect of lifetime intake and hepatitis virus infections in men and women. Am. J. Epidemiol.

[14] Hashimoto E, Taniai M, Kaneda H, Tokushige K, Hasegawa K, Okuda H, Shiratori K, Takasaki K (2004). Comparison of hepatocellular carcinoma patients with alcoholic liver disease and nonalcoholic steatohepatitis. Alcohol Clin. Exp. Res.

[15] Maheshwari S, Sarraj A, Kramer J, El-Serag HB (2007). Oral contraception and the risk of hepatocellular carcinoma. J. Hepatol.

[16] Hellerbrand C, Poppl A, Hartmann A, Scholmerich J, Lock G (2003). HFE C282Y heterozygosity in hepatocellular carcinoma: evidence for an increased prevalence. Clin. Gastroenterol. Hepatol.

[17] Merle P, Kim M, Herrmann M, Gupte A, Lefrançois L, Califano S, Trépo C, Tanaka S, Vitvitski L, Monte S, Wands JR (2005). Oncogenic role of the frizzled-7/beta-catenin pathway in hepatocellular carcinoma. J. Hepatol.

[18] Terris B, Pineau P, Bregeaud L, Valla D, Belghiti J, Tiollais P, Degott C, Dejean A (1999). Close correlation between beta-catenin gene alterations and nuclear accumulation of the protein in human hepatocellular carcinomas. Oncogene.

[19] Panga R, Tsea E, Poonb RTP (2006). Molecular pathways in hepatocellular carcinoma. Cancer Lett.

[20] Laurent-Puig P, Zucman-Rossi J (2006). Genetics of hepatocellular tumors. Oncogene.

[21] Lemmer ER, Friedman SL, Llovet JM (2006). Molecular diagnosis of chronic liver disease and hepatocellular carcinoma: the potential of gene expression pro?ling. Semin. Liver Dis.

[22] Zhao LJ, Wang L, Ren H, Cao J, Li L, Ke JS, Qi ZT (2005). Hepatitis C virus E2 protein promotes human hepatoma cell proliferation through the MAPK/ERK signaling pathway *via* cellular receptors. Exp. Cell Res.

[23] Panteva M, Korkaya H, Jameel S (2003). Hepatitis viruses and the MAPK pathway: is this a survival strategy?. Virus Res.

[24] Nagai H, Kim YS, Konishi N, Baba M, Kubota T, Yoshi-mura A, Emi M (2002). Combined hypermethylation and chromosome loss associated with inactivation of SSI-1/SOCS-1/JAB gene in human hepatocellular carcinomas. Cancer Lett.

[25] Manning JE, Harris CC, Herman JG (2001). SOCS-1, a negative regulator of the JAK/STAT pathway, is silenced by methylation in human hepatocellular carcinoma and shows growth-suppression activity. Nat. Genet.

[26] Yasuda E, Kumada T, Takai S, Ishisaki A, Noda T, Matsushima-Nishiwaki R, Yoshimi N, Kato K, Toyoda H, Kaneoka Y, Yamaguchi A, Kozawa O (2005). Attenuated phosphorylation of heat shock protein 27 correlates with tumor progression in patients with hepatocellular carcinoma. Biochem. Biophys. Res. Commun.

[27] Roberts LR, Gores GJ (2005). Hepatocellular carcinoma: Molecular pathways and new therapeutic targets. Semin. Liver Dis.

[28] Kremsdorf D, Soussan P, Paterlini-Brechot P, Brechot C (2006). Hepatitis B virus-related hepatocellular carcinoma: paradigms for viral-related human carcinogenesis. Oncogene.

[29] Lunn RM, Zhang YJ, Wang LY, Chen CJ, Lee PH, Lee CS, Tsai WY, Santella RM (1997). p53 mutations, chronic hepatitis B virus infection, and aflatoxin exposure in hepatocellular carcinoma in Taiwan. Cancer Res.

[30] Sun XY, Wang QZ, Xu RA, Xu RA, Chen L, Xiao W (2008). New Therapy of Hepatocellular Carcinoma. Molecular Gene Medicine.

[31] Tanaka S, Arii S (2009). Molecularly targeted therapy for hepatocellular carcinoma. Cancer Sci.

[32] Llovet JM, Sala M, Castells L, Suarez Y, Vilana R, Bianchi L, Ayuso C, Vargas V, Rodés J, Bruix J (2000). Randomized controlled trial of interferon treatment For advanced hepatocellular carcinoma. Hepatology.

[33] Patt YZ, Hassan MM, Lozano RD, Brown TD, Vauthey JN, Curley SA, Ellis LM (2003). Phase II trial of systemic continuous ?uorouracil and subcutaneous recombinantinterferon Alfa-2b for treatment of hepatocellular carcinoma. J. Clin. Oncol.

[34] Lin AY, Brophy N, Fisher GA, So S, Biggs C, Yock TI, Levitt L (2005). PhaseII study of thalidomide in patients with unresectable hepatocellular carcinoma. Cancer.

[35] Patt YZ, Hassan MM, Lozano RD, Nooka AK, Schnirer II, Zeldis JB, Abbruzzese JL, Brown TD (2005). Thalidomide in the treatment of patients with hepatocellular carcinoma: a phase II trial. Cancer.

[36] Zhu AX, Fuchs CS, Clark JW, Muzikansky A, Taylor K, Sheehan S, Tam K, Yung E, Kulke MH, Ryan DP (2005). A phase II study of epirubicin and thalidomide in unresectable or metastatic hepatocellular carcinoma. Oncologist.

[37] Philip PA, Mahoney MR, Allmer C, Thomas J, Pitot HC, Kim G, Donehower RC, Fitch T, Picus J, Erlichman C (2005). Phase II study of erlotinib (OSI-774) in patients with advanced hepatocellular cancer. J. Clin. Oncol.

[38] Thomas MB, Chadha R, Glover K, Wang X, Morris J, Brown T, Rashid A, Dancey J, Abbruzzese JL (2007). Phase 2 study of erlotinib in patients with unresectable hepatocellular carcinoma. Cancer.

[39] Malka D, Dromain C, Farace F, Horn S, Pignon J, Ducreux M, Boige V (2007). Bevacizumab in patients (pts) with advanced hepatocellular carcinoma (HCC): preliminary results of a phase II study with circulating endothelial cells (CEC) monitoring. J. Clin. Oncol.

[40] Zhu A, Sahani D, Tomaso E, Duda D, Sindhwani V, Yoon SS, Blaszkowsky LS, Clark JW, Ryan DP, Jain RK (2007). A phase II sutdy of sunitinib in patients with advanced hepatocellular carcinoma. J. Clin. Oncol.

[41] Lee RC, Feinbaum RL, Ambros V (1993). The *C. elegans* heterochronic gene lin-4 encodes small RNAs with antisense complementarity to lin-14. Cell.

[42] Kim VN, Nam JW (2006). Genomics of microRNA. Trends Genet.

[43] Kloosterman WP, Plasterk RHA (2006). The diverse functions of microRNAs in animal development and disease. Dev. Cell.

[44] Calin GA, Dumitru CD, Shimizu M, Bichi R, Zupo S, Noch E, Aldler H, Rattan S, Keating M, Rai K, Rassenti L, Kipps T, Negrini M, Bullrich F, Croce CM (2002). Frequent deletions and down-regulation of micro-RNA genes miR15 and miR16 at 13q14 in chronic lymphocytic leukemia. Proc. Natl. Acad. Sci. U.S.A.

[45] He L, Thomson JM, Hemann MT, Hernando-Monge E, Mu D, Goodson S, Powers S, Cordon-Cardo C, Lowe SW, Hannon GJ, Hammond SM (2005). A microRNA polycistron as a potential human oncogene. Nature.

[46] Mayr C, Hemann MT, Bartel DP (2007). Disrupting the pairing between let-7 and Hmga2 enhances oncogenic transformation. Science.

[47] Lee YS, Dutta A (2007). The tumor suppressor microRNA let-7 represses the HMGA2 oncogene. Genes Dev.

[48] Calin GA, Sevignani C, Dumitru CD, Hyslop T, Noch E, Yendamuri S, Shimizu M, Rattan S, Bullrich F, Negrini M, Croce CM (2004). Human microRNA genes are frequently located at fragile sites and genomic regions involved in cancers. Proc. Natl. Acad. Sci. U.S.A.

[49] Lu J, Getz G, Miska EA, Alvarez-Saavedra E, Lamb J, Peck D, Sweet-Cordero A, Ebert BL, Mak RH, Ferrando AA, Downing JR, Jacks T, Horvitz HR, Golub TR (2005). MicroRNA expression profiles classify human cancers. Nature.

[50] Blenkiron C, Goldstein LD, Thorne NP, Spiteri I, Chin SF, Dunning MJ, Barbosa-Morais NL, Teschendorff AE, Green AR, Ellis IO, Tavaré S, Caldas C, Miska EA (2007). MicroRNA expression profiling of human breast cancer identifies new markers of tumor subtype. Genome Biol.

[51] Michael MZ, O' Connor SM, van Holst Pellekaan NG, Young GP, James RJ (2003). Reduced accumulation of specificmicroRNAs in colorectal neoplasia. Mol. Cancer Res.

[52] Johnson SM, Grosshans H, Shingara J, Byrom M, Jarvis R, Cheng A, Labourier E, Reinert KL, Brown D, Slack FJ (2005). RAS is regulated by the let-7 microRNA family. Cell.

[53] Takamizawa J, Konishi H, Yanagisawa K, Tomida S, Osada H, Endoh H, Harano T, Yatabe Y, Nagino M, Nimura Y, Mitsudomi T, Takahashi T (2004). Reduced expression of the let-7 microRNAs in human lung cancers in association with shortened postoperative survival. Cancer Res.

[54] Eis PS, Tam W, Sun L, Chadburn A, Li Z, Gomez MF, Lund E, Dahlberg JE (2005). Accumulation of miR-155 and BIC RNA in human B cell lymphomas. Proc. Natl. Acad. Sci. U.S.A.

[55] Iorio MV, Ferracin M, Liu CG, Veronese A, Spizzo R, Sabbioni S, Magri E, Pedriali M, Fabbri M, Campiglio M, Ménard S, Palazzo JP, Rosenberg A, Musiani P, Volinia S, Nenci I, Calin GA, Querzoli P, Negrini M, Croce CM (2005). MicroRNA gene expression deregulation in human breast cancer. Cancer Res.

[56] He H, Jazdzewski K, Li W, Liyanarachchi S, Nagy R, Volinia S, Calin GA, Liu CG, Franssila K, Suster S, Kloos RT, Croce CM, Chapelle A (2005). The role of microRNA genes in papillary thyroid carcinoma. Proc. Natl. Acad. Sci. U.S.A.

[57] Murakami Y, Yasuda T, Saigo K, Urashima T, Toyoda H, Okanoue T, Shimotohno K (2006). Comprehensive analysis of microRNA expression patterns in hepatocellular carcinoma and non-tumorous tissues. Oncogene.

[58] Jiang J, Gusev Y, Aderca I, Mettler TA, Nagorney DM, Brackett DJ, Roberts LR, Schmittgen TD (2008). Association of MicroRNA expression in hepatocellular carcinomas with hepatitis infection, cirrhosis, and patient survival. Clin. Cancer Res.

[59] Ura S, Honda M, Yamashita T, Ueda T, Takatori H, Nishino R, Sunakozaka H, Sakai Y, Horimoto K, Kaneko S (2009). Differential microRNA expression between hepatitis B and hepatitis C leading disease progression to hepatocellular carcinoma. Hepatology.

[60] Calin GA, Croce CM (2006). MicroRNA Signatures in Human Cancers. Nat. Rev. Cancer.

[61] Huang YS, Dai Y, Yu XF, Bao SY, Yin YB, Tang M, Hu CX (2008). Microarray analysis of microRNA expression in hepatocellular carcinoma and non-tumorous tissues without viral hepatitis. J. Gastroenterol. Hepatol.

[62] Ji J, Shi J, Budhu A, Yu Z, Forgues M, Roessler S, Ambs S, Chen Y, Meltzer PS, Croce CM, Qin LX, Man K, Lo CM, Lee J, Ng IO, Fan J, Tang ZY, Sun HC, Wang XW (2009). MicroRNA expression, survival, and response to interferon in liver cancer. N. Engl. J. Med.

[63] Ladeiro Y, Couchy G, Balabaud C, Bioulac-Sage P, Pelletier L, Rebouissou S, Zucman-Rossi J (2008). MicroRNA profiling in hepatocellular tumors is associated with clinical features and oncogene/tumor suppressor gene mutations. Hepatology.

[64] Su H, Yang JR, Xu T, Huang J, Xu L, Yuan Y, Zhuang SM (2009). MicroRNA-101, down-regulated in hepatocellular carcinoma, promotes apoptosis and suppresses tumorigenicity. Cancer Res.

[65] Gramantieri L, Ferracin M, Fornari F, Veronese A, Sabbioni S, Liu CG, Calin GA, Giovannini C, Ferrazzi E, Grazi GL, Croce CM, Bolondi L, Negrini M (2007). Cyclin G1 is a target of miR-122a, a microRNA frequently down-regulated in human hepatocellular carcinoma. Cancer Res.

[66] Huang XH, Wang Q, Chen JS, Fu XH, Chen XL, Chen LZ, Li W, Bi J, Zhang LJ, Fu Q, Zeng WT, Cao LQ, Tan HX, Su Q (2009). Bead-based microarray analysis of microRNA expression in hepatocellular carcinoma: miR-338 is downregulated. Hepatol. Res.

[67] Zhang X, Liu S, Hu T, Liu S, He Y, Sun S (2009). Up-regulated microRNA-143 transcribed by nuclear factor kappa B enhances hepatocarcinoma metastasis by repressing fibronectin expression. Hepatology.

[68] Budhu A, Jia HL, Forgues M, Liu CG, Goldsteir D, Lam A, Zanetti KA, Ye QH, Qin LY, Croce CM, Tang ZY, Wang XW (2008). Identification of metastasis-related microRNAs in hepatocellular carcinoma. Hepatology.

[69] Love TM, Moffett HF, Novina CD (2008). Not miR-ly small RNAs: big potential for microRNAs in therapy. J. Allergy Clin. Immunol.

[70] Kota J, Chivukula RR, O'Donnell KA, Wentzel EA, Montgomery CL, Hwang HW, Chang TC, Vivekanandan P, Torbenson M, Clark KR, Mendell JR, Mendell JT (2009). Therapeutic microRNA delivery suppresses tumorigenesis in a murine liver cancer model. Cell.

[71] Suzuki HI, Yamagata K, Sugimoto K, Iwamoto T, Kato S, Miyazono K (2009). Modulation of microRNA processing by p53. Nature.

[72] Heo I, Joo C, Kim K, Ha M, Yoon MJ, Cho J, Yeom KH, Han J, Kim VN (2009). TUT4 in concert with Lin28 suppresses microRNA biogenesis through pre-microRNA uridylation. Cell.

[73] Viswanathan SR, Daley GQ, Gregory RI (2008). Selective blockade of microRNA processing by Lin28. Science.

[74] Takwi A, Li Y (2009). The p53 Pathway Encounters the MicroRNA World. Curr. Genomics.

[75] Song B, Wang Y, Kudo K, Gavin EJ, Xi Y, Ju J (2008). miR-192 Regulates dihydrofolate reductase and cellular proliferation through the p53-microRNA circuit. Clin. Cancer Res.

[76] Le MT, Teh C, Shyh-Chang N, Xie H, Zhou B, Korzh V, Lodish HF, Lim B (2009). MicroRNA-125b is a novel negative regulator of p53. Genes Dev.

[77] Chen C (2008). New development of microRNA research and role of miR-34s in p53 tumor suppressor network. In vitro. Cell. Dev. Biol. Anim.

[78] He L, He X, Lim LP, de Stanchina E, Xuan Z, Liang Y, Xue W, Zender L, Magnus J, Ridzon D, Jackson AL, Linsley PS, Chen C, Lowe SW, Cleary MA, Hannon GJ (2007). A microRNA component of the p53 tumour suppressor network. Nature.

[79] Corney DC, Flesken-Nikitin A, Godwin AK, Wang W, Nikitin AY (2007). MicroRNA-34b and MicroRNA-34c are targets of p53 and cooperate in control of cell proliferation and adhesion-independent growth. Cancer Res.

[80] Fornari F, Gramantieri L, Giovannini C, Veronese A, Ferracin M, Sabbioni S, Calin GA, Grazi GL, Croce CM, Tavolari S, Chieco P, Negrini M, Bolondi L (2009). MiR-122/cyclin G1 interaction modulates p53 activity and affects doxorubicin sensitivity of human hepatocarcinoma cells. Cancer Res.

[81] Hunt DR, Saab S (2009). Viral hepatitis in incarcerated adults, a medical and public health concern. Am J. Gastroenterol.

[82] Daniels D, Grytdal S, Wasley A (2009). Surveillance for acute viral hepatitis - United States. 2007. MMWR. Surveill. Summ.

[83] Dogantekin E, Dogantekin A, Avci D (2009). Automatic hepatitis diagnosis system based on Linear Discriminant Analysis and Adaptive Network based on Fuzzy Inference System. Expert Syst. Appl.

[84] Lavanchy D (2004). Hepatitis B virus epidemiology.; disease burden.; treatment.; and current and emerging prevention and control measures. J. Viral Hepat.,.

[85] Weiss. U (2005). Hepatitis C. Nature.

[86] Vivekanandan P, Kannangai R, Ray SC, Thomas DL, Torbenson M (2008). Comprehensive genetic and epigenetic analysis of occult hepatitis B from liver tissue samples. Clin. Infect. Dis.

[87] Guo, Li Y, Mu S, Zhang J, Yan Z (2009). Evidence that methylation of hepatitis B virus covalently closed circular DNA in liver tissues of patients with chronic hepatitis B modulates HBV replication. J. Med. Virol.

[88] Jin WB, Wu FL, Kong D, Guo AG (2007). HBV-encoded microRNA candidate and its target. Comput. Biol. Chem.

[89] Uprichard. S.L. Boyd B, Althage. A. Chisari FV (2005). Clearance of hepatitis B virus from the liver of transgenic mice by short hairpin RNAs. Proc. Natl. Acad. Sci. U.S.A.

[90] Gao YF, Yu L, Wei W, Li JB, Luo QL, Shen JL (2008). Inhibition of hepatitis B virus gene expression and replication by artificial microRNA. World J. Gastroenterol.

[91] Ely A, Naidoo T, Mufamadi S, Crowther C, Arbuthnot P (2008). Expressed anti-HBV primary microRNA shuttles inhibit viral replication efficiently *in vitro* and *in vivo*. Mol. Ther.

[92] Choo QL, Kuo G, Weiner AJ, Overby LR, Bradley DW, Houghton M (1989). Isolation of a cDNA clone derived from a blood-borne non-A.; non-B viral hepatitis genome. Science.

[93] Araújo FM, Machado-Lima A, Durham AM, Teixeira R, Oliveira G (2009). Sequence and structural analysis of the 5' noncoding region of hepatitis C virus in patients with chronic infection. J. Med. Virol.

[94] Liu Z, Yang F, Robotham JM, Tang H (2009). Critical role of cyclophilin A and its prolyl-peptidyl isomerase activity in the structure and function of the hepatitis C virus replication complex. J. Virol.

[95] Ilves H, Kaspar RL, Wang Q, Seyhan AA, Vlassov AV, Contag CH, Leake D, Johnston BH (2006). Inhibition of hepatitis C IRES-mediated gene expression by small hairpin RNAs in human hepatocytes and mice. Ann. N. Y. Acad. Sci.

[96] Kanda T, Steele R, Ray R, Ray RB (2007). Small interfering RNA targeted to hepatitis C virus 5' nontranslated region exerts potent antiviral effect. J. Virol.

[97] Gamble C, Trotard M, Le Seyec J, Abreu-Guerniou V, Gernigon N, Berrée F, Carboni B, Felden B, Gillet R (2009). Antiviral effect of ribonuclease conjugated oligodeoxynucleotides targeting the IRES RNA of the hepatitis C virus. Bioorg. Med. Chem. Lett.

[98] Pan Q, Henry SD, Metselaar H J, Scholte B, Kwekkeboom J, Tilanus HW, Janssen HL, van der Laan LJ (2009). Combined antiviral activity of interferon-alpha and RNA interference directed against hepatitis C without affecting vector delivery and gene silencing. J. Mol. Med.

[99] Jopling CL, Yi M, Lancaster AM, Lemon SM, Sarnow P (2005). Modulation of hepatitis C virus RNA abundance by a liver-specific MicroRNA. Science.

[100] Jopling CL, Schütz S, Sarnow P (2008). Position-dependent function for a tandem microRNA miR-122-binding site located in the hepatitis C virus RNA genome. Cell Host Microbe.

[101] Shan Y, Zheng J, Lambrecht RW, Bonkovsky HL (2007). Reciprocal effects of micro-RNA-122 on expression of heme oxygenase-1 and hepatitis C virus genes in human hepatocytes. Gastroenterology.

[102] Zhu Z, Wilson AT, Mathahs MM, Wen F, Brown KE, Luxon BA, Schmidt WN (2008). Heme oxygenase-1 suppresses hepatitis C virus replication and increases resistance of hepatocytes to oxidant injury. Hepatology.

[103] Henke JI, Goergen D, Zheng J, Song Y, Schüttler CG, Fehr C, Jünemann C (2008). Niepmann M.microRNA-122 stimulates translation of hepatitis C virus RNA. EMBO J.

[104] Murakami Y, Aly HH, Tajima A, Inoue I, Shimotohno K (2009). Regulation of the hepatitis C virus genome replication by miR-199a. J. Hepatol.

[105] Friedman SL (2008). Mechanisms of hepatic fibrogenesis. Gastroenterology.

[106] Seki E, De-Minicis S, Osterreicher CH, Kluwe J, Osawa Y, Brenner DA, Schwabe RF (2007). TLR4 enhances TGF-beta signaling and hepatic fibrosis. Nat. Med.

[107] Kato M, Putta S, Wang M, Yuan H, Lanting L, Nair I, Gunn A, Nakagawa Y, Shimano H, Todorov I, Rossi JJ, Natarajan R (2009). TGF-beta activates Akt kinase through a microRNA-dependent amplifying circuit targeting PTEN. Nat. Cell. Biol.

[108] Takashima M, Parsons CJ, Ikejima K, Watanabe S, White ES, Rippe RA (2009). The tumor suppressor protein PTEN inhibits rat hepatic stellate cell activation. J. Gastroenterol.

[109] Friedman SL, Bansal MB (2006). Reversal of hepatic fibrosis -- fact or fantasy?. Hepatology.

[110] Ji J, Zhang J, Huang G, Qian J, Wang X, Mei S (2009 ). Over-expressed microRNA-27a and 27b influence fat accumulation and cell proliferation during rat hepatic stellate cell activation. FEBS. Lett.

[111] Kwiecinski M, Strack I, Noetel A, Schievenbusch S, Scheffer M, Elfmova N, Dienes HP, Odenthal M (2008). MicroRNA-29: A novel antifbrogenic mediator in liver fbrogenesis. Hepatology.

[112] Lagos-Quintana M, Rauhut R, Yalcin A, Meyer J, Lendeckel W, Tuschl T (2002). Identification of tissue-specific microRNAs from mouse. Curr. Biol.

[113] Chang J, Nicolas E, Marks D, Sander C, Lerro A, Buendia MA, Xu C, Mason WS, Moloshok T, Bort R, Zaret KS, Taylor JM (2004). miR-122, a mammalian liver-specific microRNA, is processed from hcr mRNA and may downregulate the high affinity cationic amino acid transporter CAT-1. RNA Biol.

[114] Girard M, Jacqueminm E, Munnich A, Lyonnet S, Henrion-Caude A (2008 ). miR-122, a paradigm for the role of microRNAs in the liver. J. Hepatol..

[115] Jacob JR, Sterczer A, Toshkov IA, Yeager AE, Korba BE, Cote PJ, Buendia MA, Gerin JL, Tennant BC (2004 ). Integration of woodchuck hepatitis and N-myc rearrangement determine size and histologic grade of hepatic tumors. Hepatology.

[116] Kutay H, Bai S, Datta J, Motiwala T, Pogribny I, Frankel W, Jacob ST, Ghoshal K (2006). Downregulation of miR-122 in the rodent and human hepatocellular carcinomas. J. Cell. Biochem.

[117] Meng F, Hensonm R, Wehbe-Janek H, Ghoshal K, Jacob ST, Patel T (2007). MicroRNA-21 regulates expression of the PTEN tumor suppressor gene in human hepatocellular cancer. Gastroenterology.

[118] Tsai WC, Hsu PW, Lai TC, Chau GY, Lin CW, Chen CM, Lin CD, Liao YL, Wang JL, Chau YP, Hsu MT, Hsiao M, Huang HD, Tsou AP (2009). MicroRNA-122, a tumor suppressor microRNA that regulates intrahepatic metastasis of hepatocellular carcinoma. Hepatology.

[119] Esau C, Davis S, Murray SF, Yu XX, Pandey SK, Pear M, Watts L, Booten SL, Graham M, McKay R, Subramaniam A, Propp S, Lollo BA, Freier S, Bennett CF, Bhanot S, Monia BP (2006). miR-122 regulation of lipid metabolism revealed by *in vivo* antisense targeting. Cell Metab.

[120] Scherr M, Venturini L, Battmer K, Schaller-Schoenitz M, Schaefer D, Dallmann I, Ganser A, Eder M (2007). Lentivirus-mediated antagomir expression for specific inhibition of miRNA function. Nucleic Acids Res.

[121] Krützfeldt J, Rajewsky N, Braich R, Rajeev KG, Tuschl T, Manoharan M, Stoffel M (2005). Silencing of microRNAs *in vivo* with 'antagomirs'. Nature.

[122] Gentner B, Schira G, Giustacchini A, Amendola M, Brown BD, Ponzoni M, Naldini L (2009). Stable knockdown of microRNA *in vivo* by lentiviral vectors. Nat. Method.

[123] Haraguchi T, Ozaki Y, Iba H (2009). Vectors expressing efficient RNA decoys achieve the long-term suppression of specific microRNA activity in mammalian cells. Nucleic Acids Res.

[124] Ye QH, Qin LX, Forgues M, He P, Kim JW, Peng AC, Simon R, Li Y, Robles AI, Chen Y, Ma ZC, Wu ZQ, Ye SL, Liu YK, Tang ZY, Wang XW (2003). Predicting hepatitis B virus-positive metastatic hepatocellular carcinomas using gene expression profiling and supervised machine learning. Nat. Med.

[125] Sun BS, Dong QZ, Ye QH, Sun HJ, Jia HL, Zhu XQ, Liu DY, Chen J, Xue Q, Zhou HJ, Ren N, Qin LX (2008). Lentiviral-mediated miRNA against osteopontin suppresses tumor growth and metastasis of human hepatocellular carcinoma. Hepatology.

[126] Li S, Fu H, Wang Y, Tie Y, Xing R, Zhu J, Sun Z, Wei L, Zheng X (2009). MicroRNA-101 regulates expression of the v-fos FBJ murine osteosarcoma viral oncogene homolog (FOS) oncogene in human hepatocellular carcinoma. Hepatology.

[127] Sieghart W, Losert D, Strommer S, Cejka D, Schmid K, Rasoul-Rockenschaub S, Bodingbauer M, Crevenna R, Monia BP (2006). Mcl-1 overexpression in hepatocellular carcinoma: a potential target for antisense therapy. J. Hepatol.

[128] Lin CJ, Gong HY, Tseng HC, Wang WL, Wu JL (2008). miR-122 targets an anti-apoptotic gene, Bcl-w, in human hepatocellular carcinoma cell lines. Biochem. Biophys. Res. Commun.

[129] Bai S, Nasser MW, Wang B, Hsu SH, Datta J, Kutay H, Yadav A, Nuovo G, Kumar P, Ghoshal K (2009). MicroRNA-122 inhibits tumorigenic properties of hepatocellular carcinoma cells and sensitizes these cells to sorafenib. J. Biol. Chem.

[130] Coulouarn C, Factor VM, Andersen JB, Durkin ME, Thorgeirsson SS (2009). Loss of miR-122 expression in liver cancer correlates with suppression of the hepatic phenotype and gain of metastatic properties. Oncogene.

[131] Ma L, Liu J, Shen J, Liu L, Wu J, Li W, Luo J, Chen Q, Qian C (2010). Expression of miR-122 mediated by adenoviral vector induces apoptosis and cell cycle arrest of cancer cells. Cancer Biol. Ther.

[132] Wong QW, Lung RW, Law PT, Lai PB, Chan KY, To KF, Wong N (2008). MicroRNA-223 is commonly repressed in hepatocellular carcinoma and potentiates expression of Stathmin1. Gastroenterology.

[133] Connolly E, elegari M, Landgraf P, Tchaikovskaya T, Tennant BC, Slagle BL, Rogler LE, Zavolan M, Tuschl T, Rogler CE (2008). Elevated expression of the miR-17-92 polycistron and miR-21 in hepadnavirus-associated hepatocellular carcinoma contributes to the malignant phenotype. Am. J. Pathol.

[134] Medina R, Zaidi SK, Liu CG, Stein JL, van Wijnen AJ, Croce CM, Stein GS (2008). MicroRNAs 221 and 222 bypass quiescence and compromise cell survival. Cancer Res.

[135] Fornari F, Gramantieri L, Ferracin M, Veronese A, Sabbioni S, Calin GA, Grazi GL, Giovannini C, Croce CM, Bolondi L, Negrini M (2008). MiR-221 controls CDKN1C/p57 and CDKN1B/p27 expression in human hepatocellular carcinoma. Oncogene.

[136] Gramantieri L, Fornari F, Ferracin M, Veronese A, Sabbioni S, Calin GA, Grazi GL, Croce CM, Bolondi L, Negrini M (2009). MicroRNA-221 targets Bmf in hepatocellular carcinoma and correlates with tumor multifocality. Clin. Cancer Res.

[137] Pineau P, Volinia S, McJunkin K, Marchio A, Battiston C, Terris B, Mazzaferro V, Lowe SW, Croce CM, Dejean A (2010). miR-221 overexpression contributes to liver tumorigenesis. Proc. Natl. Acad. Sci. U.S.A.

[138] Garofalo M, Di Leva G, Romano G, Nuovo G, Suh SS, Ngankeu A, Taccioli C, Pichiorri F, Alder H, Secchiero P, Gasparini P, Gonelli A, Costinean S, Acunzo M, Condorelli G, Croce CM (2009). miR-221&222 regulate TRAIL resistance and enhance tumorigenicity through PTEN and TIMP3 downregulation. Cancer Cell.

[139] Bazan-Peregrino M, Seymour LW, Harris AL (2007). Gene therapy targeting to tumor endothelium. Cancer Gene Ther.

[140] Waehler R, Russell SJ, Curiel DT (2007). Engineering targeted viral vectors for gene therapy. Nat. Rev. Genet.

[141] Barber GN (2005). VSV-tumor selective replication and protein translation. Oncogene.

[142] Xu RA, Xiao WD, Lu H (2009). A novel cell specific microRNA binding sequence gene: hAAVmir for gene therapy. C.N. Patent 101,532,024.

[143] Thomas CE, Ehrhardt A, Kay MA (2003). Progress and problems with the use of viral vectors for gene therapy. Nat. Rev. Genet.

[144] De Geest BR, Van Linthout SA, Collen D (2003). Humoral immune response in mice against a circulating antigen induced by adenoviral transfer is strictly dependent on expression in antigen-presenting cells. Blood.

[145] Brown BD, Venneri MA, Zingale A, Sergi Sergi L, Naldini L (2006). Endogenous microRNA regulation suppresses transgene expression in hematopoietic lineages and enables stable gene transfer. Nat. Med.

[146] Brown BD, Cantore A, Annoni A, Sergi LS, Lombardo A, Della Valle P, D'Angelo A, Naldini L (2007). A microRNA-regulated lentiviral vector mediates stable correction of hemophilia B mice. Blood.

[147] Wolff LJ, Wolff JA, Sebestyén MG (2009). Effect of tissue-specific promoters and microRNA recognition elements on stability of transgene expression after hydrodynamic naked plasmid DNA delivery. Hum. Gene Ther.

[148] Brown BD, Gentner B, Cantore A, Colleoni S, Amendola M, Zingale A, Baccarini A, Lazzari G, Galli C, Naldini L (2007). Endogenous microRNA can be broadly exploited to regulate transgene expression according to tissue, lineage and differentiation state. Nat. Biotechnol.

[149] Ylösmäki E, Hakkarainen T, Hemminki A, Visakorpi T, Andino R, Saksela K (2008). Generation of a conditionally replicating adenovirus based on targeted destruction of E1A mRNA by a cell type-specific MicroRNA. J. Virol.

[150] Cawood R, Chen HH, Carroll F, Bazan-Peregrino M, van Rooijen N, Seymour LW (2009). Use of tissue-specific microRNA to control pathology of wild-type adenovirus without attenuation of its ability to kill cancer cells. PLoS. Pathog.

[151] Bell JC, Kirn D (2008). MicroRNAs fine-tune oncolytic viruses. Nat. Biotechnol.

[152] Suzuki T, Sakurai F, Nakamura S, Kouyama E, Kawabata K, Kondoh M, Yagi K, Mizuguchi H (2008). miR-122a-regulated expression of a suicide gene prevents hepatotoxicity without altering antitumor effects in suicide gene therapy. Mol. Ther.

[153] Wang QZ, Xu W, Habib N, Xu R (2009). Potential uses of microRNA in lung cancer diagnosis, prognosis, and therapy. Curr. Cancer Drug Targets.

[154] Edge RE, Falls TJ, Brown CW, Lichty BD, Atkins H, Bell JC (2008 ). A let-7 MicroRNA-sensitive vesicular stomatitis virus demonstrates tumor-specific replication. Mol. Ther.

[155] Lee CY, Rennie PS, Jia WW (2009). MicroRNA regulation of oncolytic herpes simplex virus-1 for selective killing of prostate cancer cells. Clin. Cancer Res.

